# Supplementary figures and images for: The IL-10GFP (VeRT-X) mouse strain is not suitable for the detection of IL-10 production by granulocytes during lung inflammation
Source: PLoS One. 2021 May 12;16(5):e0247895. doi: 10.1371/journal.pone.0247895 (PMC8115804; doi:10.1371/journal.pone.0247895)

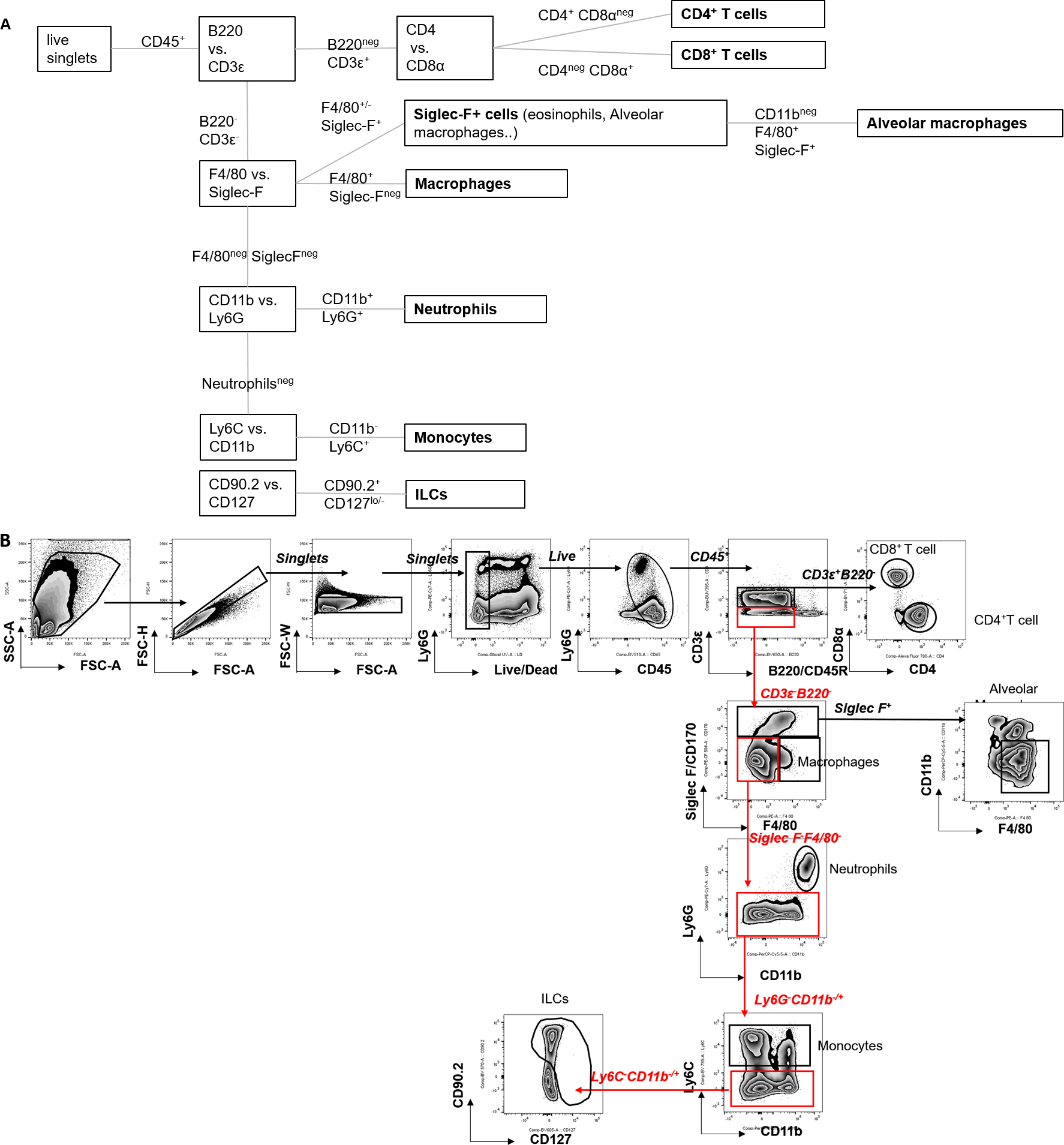

Supplement: S1 Fig — A graphic outline (A) and exemplary graphs (B) are given to illustrate the gating strategy employed to identify CD4+ T cells (CD45+ CD45R/B220- CD3ε+ CD4+ CD8α- CD127lo), CD8+ T cells (CD45+ CD45R/B220- CD3ε+ CD4- CD8α+), macrophages (CD45+ CD45R/B220- CD3ε- Siglec-F- F4/80+), Siglec-F+ cells (eosinophils, alveolar macrophages; CD45+ CD45R/B220- CD3ε- Siglec-F+ F4/80-/+), neutrophils (CD45+ CD45R/B220- CD3ε- Siglec-F- F4/80- Ly6G+ CD11b+), monocytes (CD45+ CD45R/B220- CD3ε- Siglec-F- F4/80- Ly6G- Ly6C+ CD11b+), and ILCs in the inflamed lung. (TIF) [file pone.0247895.s001.tif]

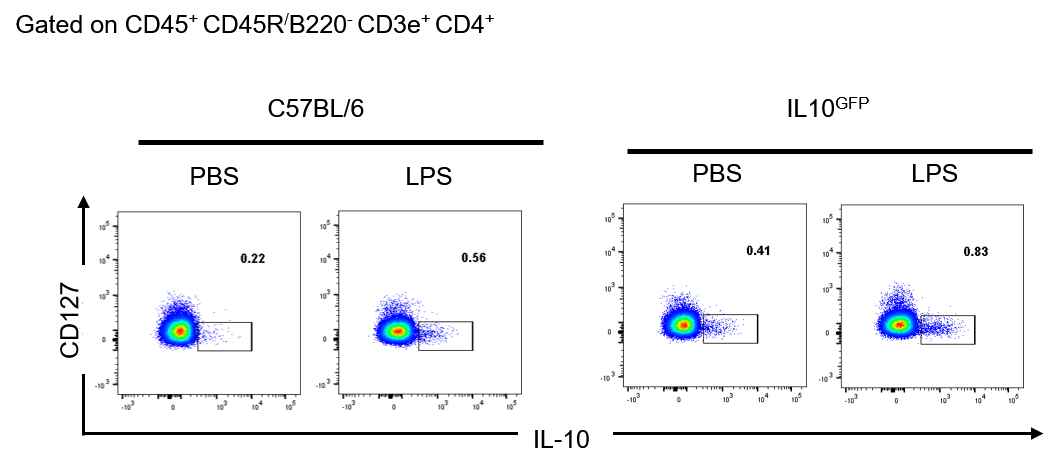

Supplement: S2 Fig — Representative flow cytometric dot plots showing the IL-10 staining (ICCS) in splenic CD4+ T cells. (TIF) [file pone.0247895.s002.tif]

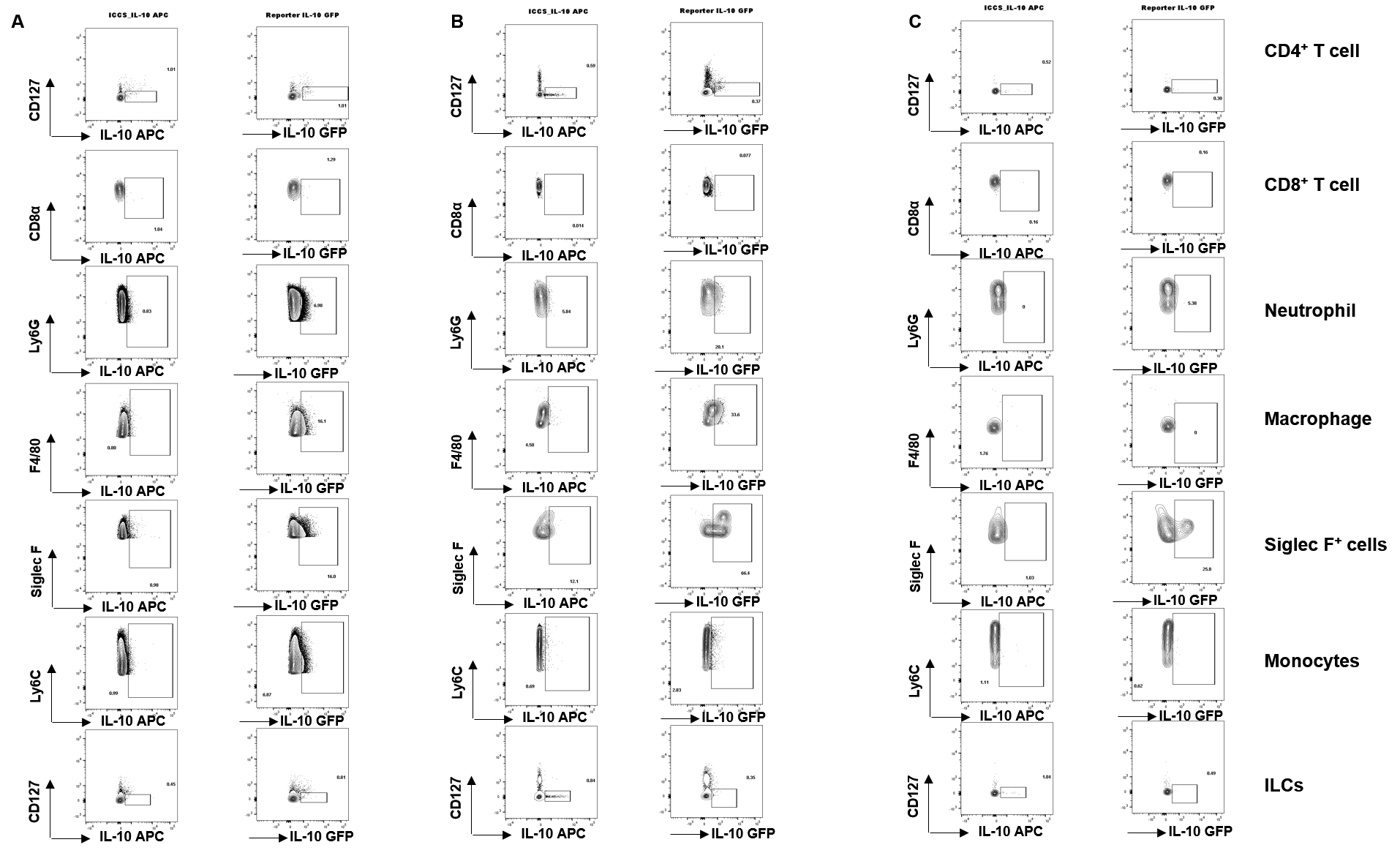

Supplement: S3 Fig — C57BL/6 and IL-10GFP mice were challenged three times (d0, d1, d2) with either PBS or 10 μg LPS per mouse via aspiration. 16–18 hours after the last administration, the single cell suspension from (A) lungs, (B) spleens, and (C) mLNs were stained for CD4+ T cells (CD45+ CD45R/B220- CD3ε+ CD4+ CD8α-), CD8+ T cells (CD45+ CD45R/B220- CD3ε+ CD4- CD8α+), macrophages (CD45+ CD45R/B220- CD3ε- Siglec-F- F4/80+), Siglec-F+ cells (eosinophils, alveolar macrophages; CD45+ CD45R/B220- CD3ε- Siglec-F+ F4/80-/+), neutrophils (CD45+ CD45R/B220- CD3ε- Siglec-F- F4/80- Ly6G+ CD11b+), monocytes (CD45+ CD45R/B220- CD3ε- Siglec-F- F4/80- Ly6G- Ly6C+ CD11b+), and ILCs (CD45+ CD45R/B220- CD3ε- Siglec-F- F4/80- Ly6G- CD90.2+ CD127lo/-). The expression of IL-10 was measured either by intracellular IL-10 staining (left panel) or GFP—expression (right panel). (TIF) [file pone.0247895.s003.tif]

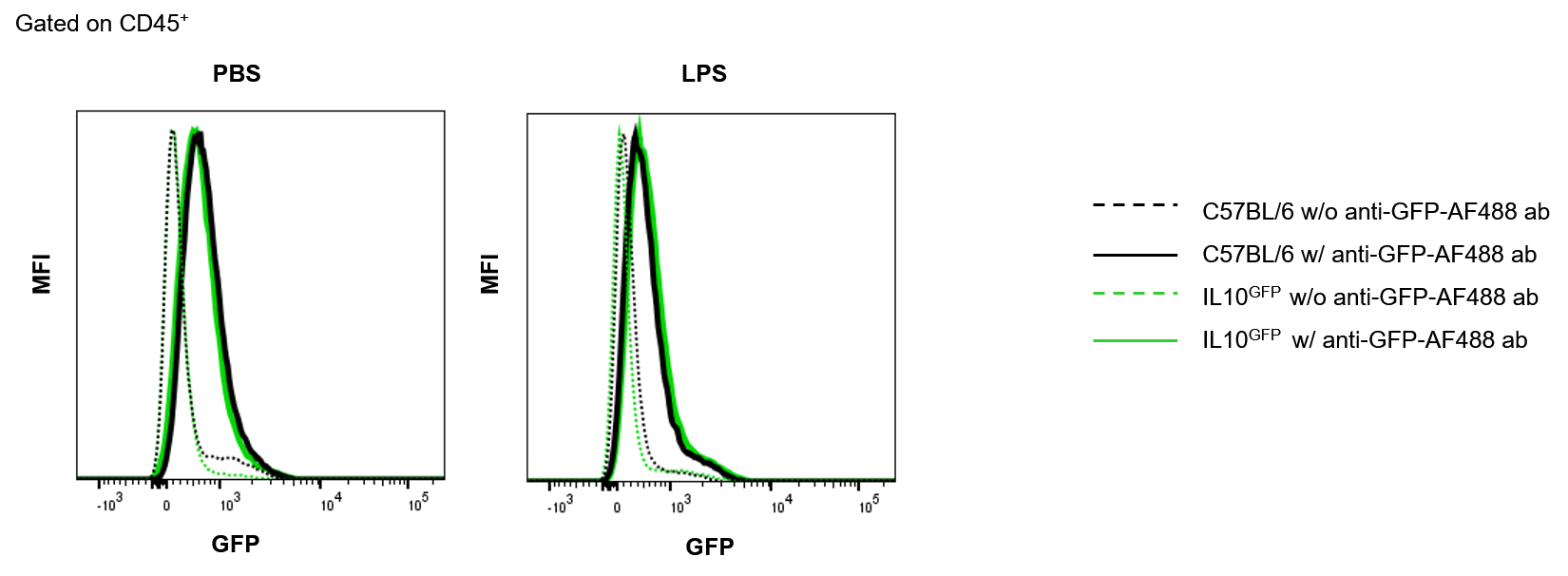

Supplement: S4 Fig — Representative histograms from the spleen of PBS (left) or LPS (right) challenged C57BL/6 and IL-10GFP mouse, demonstrating the overall GFP-signal detected with or without labelling with secondary αGFP-AF488. (TIF) [file pone.0247895.s004.tif]

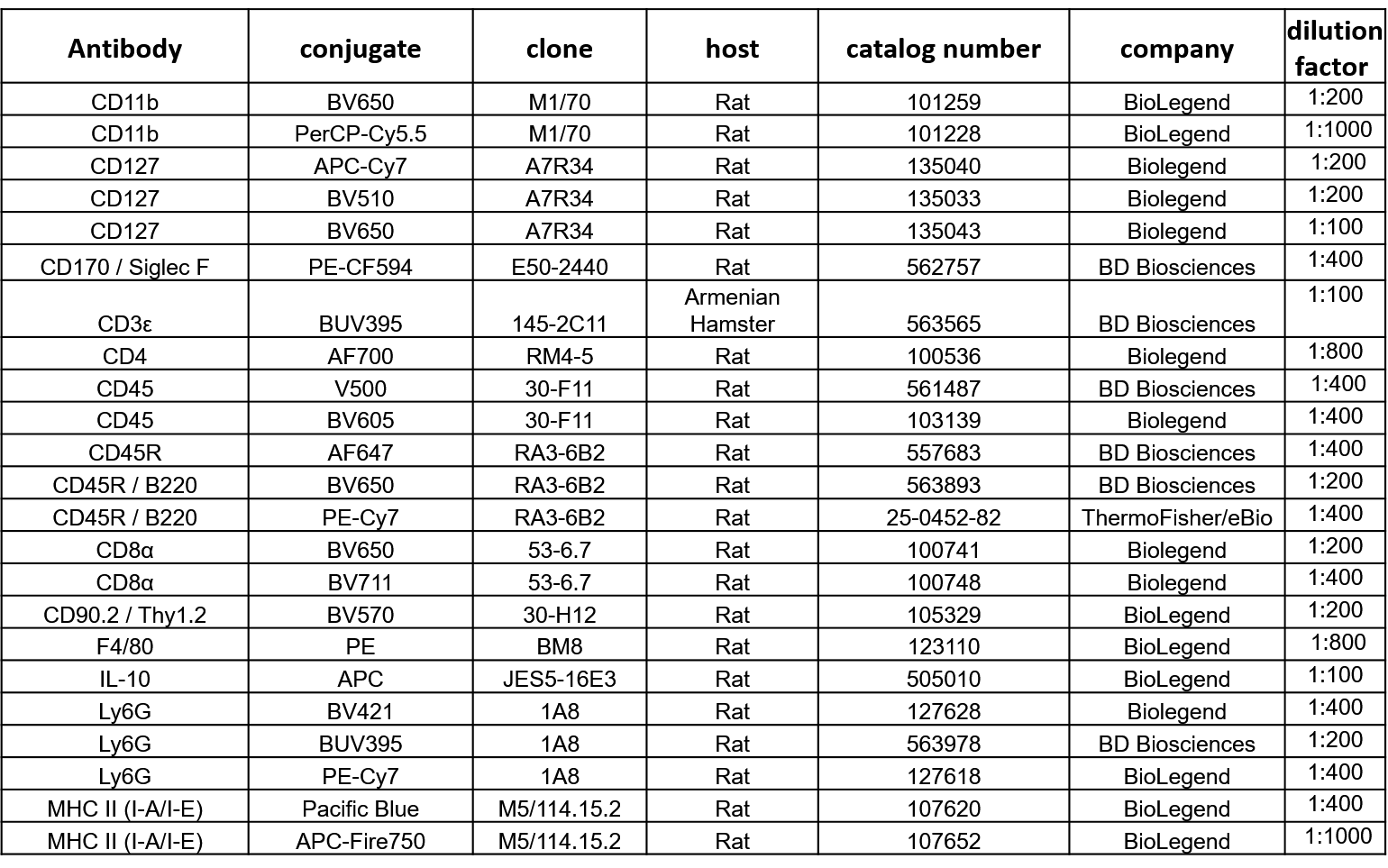

Supplement: S1 Table — (TIF) [file pone.0247895.s005.tif]
