# Supplementary material for: The IL-10GFP (VeRT-X) mouse strain is not suitable for the detection of IL-10 production by granulocytes during lung inflammation
Source: PLoS One. 2021 May 12;16(5):e0247895. doi: 10.1371/journal.pone.0247895 (PMC8115804; doi:10.1371/journal.pone.0247895)
Supplement: Attachment — Submitted filename: Point-to-point response_Ozkan et al.pdf [file pone.0247895.s006.pdf]

Asst. Prof. Gerhard Wingender Ph.D.
Izmir Biomedicine and Genome Center (IBG)
Dokuz Eylul University Health Campus
35340 Balcova/Izmir
Turkey
Email: gerhard.wingender@ibg.edu.tr

29 March 2021

RESPONSE TO THE REWIEVERS & EDITORS:

We thank the reviewers for their time and efforts in their thoughtful and thorough review, which helped us to improve the manuscript. Enclosed please find the revised version of the manuscript that takes into account the reviewers' comments and suggestions, and that highlights the important changes in the text in yellow. Please find below our point-to-point reply to the issues raised.

Editors

1. Please ensure that your manuscript meets PLOS ONE's style requirements, including those for file naming.

Response: We followed PLOS ONE's guidelines to our best knowledge and made the appropriated changes.

2. We note that you have included the phrase "data not shown" in your manuscript. PLOS does not permit references to inaccessible data. We require that authors provide all relevant data within the paper, Supporting Information files, or in an acceptable, public repository. Please add a citation to support this phrase or upload the data that corresponds with these findings to a stable repository (such as Figshare or Dryad) and provide and URLs, DOIs, or accession numbers that may be used to access these data. Or, if the data are not a core part of the research being presented in your study, we ask that you remove the phrase that refers to these data.

Response: We did remove this comment and the related statement.

3. Please upload a new copy of Supporting Information Figure 2 as the detail is not clear.

Response: We updated the figure now with a version of higher resolution.

4. Please review your reference list to ensure that it is complete and correct. If you have cited papers that have been retracted, please include the rationale for doing so in the manuscript text, or remove these references and replace them with relevant current references. Any changes to the reference list should be mentioned in the rebuttal letter that accompanies your revised manuscript. If you need to cite a retracted article, indicate the article's retracted status in the References list and also include a citation and full reference for the retraction notice.

Response: The reference list is complete and correct to the best of our knowledge. We checked the references on PubMed and none of them where flagged as retracted.

Reviewer #1

Ozkan et al describe a potential caveat of utilising IL-10 reporter mice (VeRT-X) to identify myeloid-derived IL-10 production during an experimental model of LPS-induced lung inflammation. The manuscript is well written, and the data is presented in a concise manner. The following comments are suggestions which I believe may add clarity to the overall message of the manuscript.

Major comments:

1. Can the authors comment on how the VeRT-X IL-10 reporter line and their results compare to any of the other eight IL-10 reporter lines, described by Bouabe 2012 (https://doi.org/10.1111/j.1365-3083.2012.02695.x)?

Response: As we only analysed the VeRT-X IL-10 reporter mouse line, we cannot make claims about other reporter lines and refrained from doing so in the text. However, the loss in sensitivity for myeloid cells we observed was due to the increased autofluorescence in the 'channel' used to measure GFP, i.e. 505 nm - 550 nm, and was also observed in C57BL/6 wild type mice. Therefore, as we suggested in the discussion, we would anticipate that other GFP-based reporter lines would be affected in a similar fashion. Looking at IL-10-reporter lines using fluorescent proteins, this would be relevant for the *tiger* (Kamanaka *et al.* 2006, PMID: 17137799), the B-Green (Neves *et al.* 2010, 21093317), and ITIG (Bouabe *et al.* 2011, 21844394) mouse lines. Two other IL-10 reporter lines, using the fluorescent protein YFP were reported: the IL-10^{eYFP} (Calado *et al.* 2006, 17015721) and $IL10^{Venus}$ (Atarashi *et al.* 2011, 21205640) lines. GFP's emission peak is at around 507 nm, whereas YFP/Venus's emission peak is around 527/528 nm. Nonetheless, YFP is usually also detected in the common 'FITC channel' (505 nm - 550 nm). Therefore, we expect that the YFP-signal would be impacted as well by the increased myeloid-specific autofluorescence during lung inflammation that we report here. However, we feel that discussing this YFP aspect would be off topic for our manuscript.

2. The original paper describing the VeRT-X mice (Madan et al., 2009) shows IL-10 GFP expression by B cells. Have the authors measured B cell derived IL-10 GFP levels in their model of LPS-induced lung inflammation?

Response: We did analyse IL10 in B cells as well, but did notice neither a strong IL-10 production by B cells in our experiments, nor a significant difference between the signal when measured via the $IL10^{GFP}$ - reporter or via intracellular cytokine

staining (**Figure A**). These data are for the reviewer's perusal, as we do not propose to include these negative data.

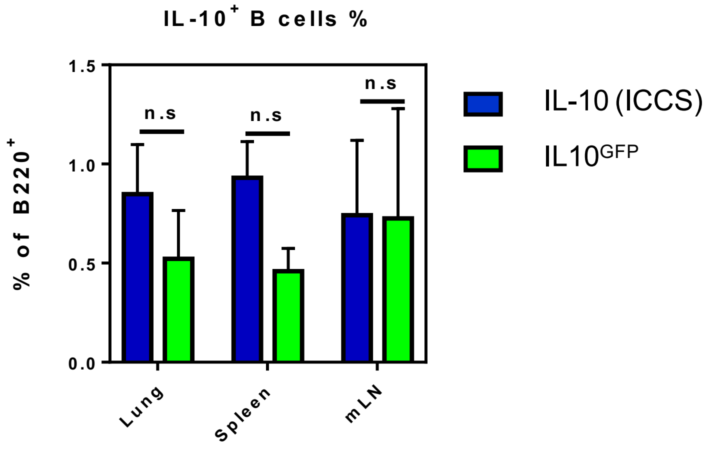

Figure A. IL-10 expression in B cells: C57BL/6 and IL-10^{GFP} mice were challenged three times (d0, d1, d2) with either PBS or 10 µg LPS per mouse via aspiration. 16-18 hours after the last administration, the single cell suspension from lungs, spleens, and mLNs were stained for B cells (CD45^{+} CD45R/B220^{+} CD3ε^{-}) and the expression of IL-10 was measured either by GFP^{+} or by intracellular IL-10 as indicated. The graph shows combined data from two independent experiments (PBS: n = 6 mice/group, LPS: n = 7-9 mice/group in total). n.s. = not significant.

3. It would be helpful to provide representative FACS plots as part of Supplementary Figure 1, to complement the gating strategy.

Response: We are happy to include this information, which is now provide in the supplementary figure 1B.

4. Figure 2 nicely illustrates IL-10 detection by ICCS vs GFP. It would be helpful to show representative FACS plots as part of this figure or as a Supplementary figure.

Response: We added a new supplementary figure (supplementary figure 3) with representative dot plots for all cell types and organs represented in figure 2.

5. The figure legend for Figure 2 states: "The graph shows combined data from two independent experiments (PBS: n = 6 mice/group. LPS: n=7-9 mice/group)". Can the authors confirm whether 6-9 mice/group were used in each individual experiment? Or whether the 6-9mice/group refers to the pooled data shown in Figure 2?

Response: The provided numbers represent the total mice for both experiments together. We added 'in total' to the text to clarify this point.

Minor comments:

Line 31: please remove 's' from inflammations

Line 49: please change where to were

Response: We thank the reviewer for pointing this out to us. The text was adjusted accordingly.

Line 54: please insert reference 7 after myeloid cells.

Response: We thank the reviewer for pointing this out to us. The reference was moved at the end of the sentence in the revised version of the manuscript.

Line 54: please check reference 8, since the autofluorescence of myeloid cells was not mentioned in this reference.

Response: We refer to the figures 1B and 2B in Mitchell *et al.* (PMID: 20534703), which show the autofluorescence in the 'GFP-channel', called 'V525' therein, in several splenic myeloid populations. Therefore, we think that this is an appropriate reference for the point made.

Line 90-91: please clarify why data is not shown from WT controls. It might be informative to show this data as a supplementary figure.

Response: After consideration, we feel the point we want to make is easier understood with the αGFP-AF488-Ab data (Supplementary figure 3) alone. Therefore, we removed the statement related 'data not shown' comment in the revised version of the manuscript.

Line 103: please remove 's' from inflammations

Line 108: please remove 'also'

Line 110: please change 'analysis' to analyzing

Line 114: please remove 's' from inflammations

Line 145: please add 'd' to purchase

Line 318: please superscript IL10GFP

Line 321 – 322: please superscript CD45+ CD45R/B220- CD3- Siglec-F+ F4/80+ CD11b- and IL10GFP

Response: We thank the reviewer for pointing these out to us. The text was changed as recommended.

Reviewer #2

In this paper, the authors have proposed that IL-10 GFP reporter strain is not an appropriate model to detect IL-10 production by granulocytes. Using C57BL/6 and IL-10 GFP mice and by employing flowcytometry, the authors show that GFP signal seen in granulocytes post LPS challenge is not the actual GFP signal due to IL-10 production. Rather, it is attributable to autofluorescence. I think the authors have used proper controls in the study and results from the study illustrates important technical caveat in using GFP reporter mice for analysing myeloid cells. Overall, the study is good.

However, I have few minor suggestions:

1. Line 31: Please change " during inflammations" to during inflammation. Please make this change elsewhere as well.

Response: We thank the reviewer for pointing this out to us. The text was adjusted accordingly.

2. Line 39-40: Please rephrase this sentence as the meaning is not clear.

Response: We rephrased the sentence in the hope to increase clarity.

3. Line 49: Please change "lines where" to lines were.

Response: We thank the reviewer for pointing this out to us. The text was adjusted accordingly.

4. Line 54: Please give reference when you discuss that IL-10 GFP strain was reported to enable identification of IL-10+ myeloid cells.

Response: We thank the reviewer for pointing this out to us. The reference was moved at the end of the sentence in the revised version of the manuscript.

5. Line 110: Please change "analysis" to analysing.

6. Line 170: Change "where" to were.

Response: We thank the reviewer for pointing these out to us. The text was changed as recommended.

7. Line 277-279 and 295-296: Please rephrase these two sentences as they seem incomplete.

Response: We rephrased the two sentences to improve their clarity.

8. Line 337-339: I think this sentence " C) Representative histograms from the spleen of PBS-------" is not required as it is a repetition of Supplementary Figure 3 legend.

Response: We thank the reviewer for pointing this out to us. The duplicated text was removed in this revised version of the manuscript.

9. Please check the overall grammar and sentence structure throughout the text.

Response: The thoroughly reviewed the text once more to our best knowledge.

We believe that we have fully addressed all the reviewers' concerns and hope that our revised manuscript is now suitable for publication in *PLOS ONE*.

Please do not hesitate to contact me if you had any questions.

Yours sincerely,

G. Wingender

Gerhard Wingender
